# Impact of Financial Incentives on Intimate Partner Violence for Women Living with HIV Initiating Antiretroviral Therapy

**DOI:** 10.1007/s10461-025-04621-1

**Published:** 2025-02-17

**Authors:** Rebecca Hémono, Emmanuel Katabaro, Babuu Joseph, Hamza Maila, Janeth Msasa, Kassim Hassan, Solis Winters, Ndola Prata, William H. Dow, Prosper Njau, Amon Sabasaba, Sandra I. McCoy

**Affiliations:** 1 School of Public Health, University of California, Berkeley, Berkeley, CA USA; 2 Health for a Prosperous Nation, Dar es Salaam, Tanzania; 3 National AIDS Control Programme, Ministry of Health, Dar es Salaam, Tanzania

**Keywords:** IntimatePartner Violence, Gender-based Violence, Financial Incentives, Economic Support, HIV

## Abstract

Financial incentives have demonstrated effectiveness in improving outcomes for people living with HIV; however, little is known about their impact on intimate partner violence (IPV) in this vulnerable population. A cluster-randomized trial investigating financial incentives and viral suppression was conducted in the Lake Zone of Tanzania (clinicaltrials.gov: NCT0420135). Between May 2021-March 2022, 32 clinics were randomized 1:1 to receive the standard of care (SOC) or to offer 22,500 TZS (~$10 USD) to individuals initiating antiretroviral therapy monthly for up to 6 months, conditional on clinic attendance. An intention-to-treat analysis was conducted to examine the effect of financial incentives on IPV among partnered women at 6 months (*n* = 494), with prevalence differences (PD) and 95% confidence intervals (CI) estimated using generalized estimating equations. Subgroup analyses were conducted among women partnered at 6 months only and women partnered at both baseline and 6 months. Past 6-month IPV was reported by 241 (48.8%) partnered women. Emotional IPV was the most common form of violence (47.8%), followed by physical (7.5%) and sexual IPV (5.1%). There were no significant differences in past 6-month IPV between arms in the primary (49.6% SOC vs. 48.1% incentive, PD: -0.02, 95% CI: -0.18, 0.15) or subgroup analyses. These findings suggest that small, short-term financial incentives used to improve HIV treatment outcomes do not significantly alter IPV risks, however with the width of the CIs it is not possible to rule out small and moderate-sized effects. Further research among larger samples is needed as financial incentive programs are brought to scale for people living with HIV.

**Supplementary Information:**

The online version contains supplementary material available at 10.1007/s10461-025-04621-1.

## Introduction

Despite large improvements in uptake and adherence to antiretroviral therapy (ART) following the 95-95-95 targets [1, 2], access to HIV care in sub-Saharan Africa remains stymied by pervasive structural barriers including stigma, poverty, prohibitive norms, and gender-inequality [3–6], which hinder efforts from reaching their full potential. Women experiencing intimate partner violence (IPV), who represent a large population of people living with HIV [7], experience exacerbated barriers to HIV care such as interference from partners, verbal threats and intimidation, limited freedom of movement, and lack of financial autonomy and ability to pay for transport for clinic visits [7–12]. In turn, women in violent partnerships experience higher risk of poor HIV outcomes such as disengagement from care and virologic failure, which can heighten the risk of onward transmission [13, 14]. 

Interventions grounded in economic theory and behavioral economics such as financial incentives have been used for more than two decades to motivate engagement with HIV prevention and treatment services [15–18]. Theories from psychology and economics posit that offering these forms of economic support can influence behaviors through extrinsic motivation (e.g., encouraging care-seeking through offering an incentive to visit a health facility) and addressing present-bias (i.e., increasing shorter-term rewards, given the tendency to respond more to immediate rewards relative to longer-term benefits) [19, 20]. Furthermore, there is evidence suggesting that incentives do not have to be large to be effective and that frequent, small incentives can be a sufficient behavioral nudge to change and sustain healthy behaviors [19, 21]. 

Several rigorous studies have demonstrated the effectiveness of financial incentives in increasing testing and strengthening linkage, adherence, and retention in HIV care [21–26], and UNAIDS recommends the provision of incentives for people living with HIV [27]. As a result, financial incentives have increasingly been adopted by researchers and practitioners as a strategy to improve HIV outcomes and have the potential to be scaled up and implemented in a wider variety of settings, including in combination with other behavioral interventions [28]. However, the existing literature on economic support and their effects on women’s safety in intimate relationships is limited and mixed [29, 30], and little is known about whether and how women living with HIV are uniquely affected, particularly when financial incentives are provided as small, behavioral nudges.

Some evidence among women in the general population suggests that providing economic support can lead to reductions in IPV [31, 32]. A randomized trial in Kenya comparing the effects of unconditional cash payments for women and men on IPV found that providing women with cash was associated with significant reductions in both physical and sexual violence and cash transfers for men decreased physical violence [31]. The JUNTOS program in Peru, which distributed payments to mothers and pregnant women conditional on school attendance and healthcare visits, showed that physical IPV decreased by 25% for program participants [33]. The Oportunidades social protection and cash transfer program in Mexico also demonstrated large declines in IPV, with physical violence reduced by 40% among beneficiaries [34]. 

However, providing economic support has also been shown to increase violence in certain contexts, with impacts varying according to the socio-demographic characteristics of recipients [34]. For example, a subsequent analysis of Oportunidades revealed that although women beneficiaries experienced less physical violence, they were more likely to experience emotional violence by their partners including threats [34]. Aggressive behaviors also increased when large transfers were received by households with uneducated husbands, particularly when married to younger women [35]. The Bono Solidario program in Ecuador demonstrated differential effects depending on the couples’ educational status, with decreased emotional violence for women who had more than a primary school education and increased emotional violence for women who had the same education or more than her partner [36]. 

There are several pathways which may explain why economic interventions can mitigate risks of violence in some cases and exacerbate risks in others. Some hypothesize that providing women with economic resources can reduce violence by decreasing stress related to poverty or financial uncertainty, improving women’s bargaining power, and empowering women to leave violent relationships [37–39]. On the contrary, as women’s economic autonomy increases, male partners might feel threatened and perpetrate violence in an effort to regain power and control [40–42]. The type of intervention, size of the financial reward, implementation approach, and beneficiary population are important factors which may influence these pathways [30, 43]. However, the interplay of these factors is complex, and there is limited understanding of how and why certain groups benefit from certain economic interventions and others experience increased risks.

Given the inconsistent evidence on economic support and IPV and the growing number of financial incentive programs implemented for HIV care, it is imperative to examine how offering incentives affects violence among women living with HIV, a vulnerable population who experience heightened IPV risks compared to the general population [44] and thus may need additional safeguarding mechanisms to protect from any unintended harms. To address this gap, we investigated the relationship between small, short-term financial incentives and IPV among women living with HIV receiving incentives to motivate adherence to HIV treatment.

## Methods

### Study Overview and Population

We analyzed 6-month data from a cluster-randomized trial which evaluated the effectiveness of 6 months of financial incentives for improving retention in HIV care among individuals initiating ART. The study was conducted at 32 HIV clinics across four regions (Mwanza, Kagera, Geita, Shinyanga) in the Lake Zone of Tanzania (clinicaltrials.gov: *NCT0420135*). The estimated HIV prevalence among adults in these regions ranges from 4.7–5.7% [45]; lifetime experiences of IPV were reported by 38–53% of women ages 15–49 [46]. The primary outcome of the trial was viral suppression at 12 months, 6 months after the financial incentive intervention concluded [47]. 

Eligible clinics were randomized in a 1:1 ratio to receive the standard of care (SOC), per the National Guidelines for HIV care in Tanzania [48], or to offer to all ART initiates (initiated care within 30 days of enrollment) a financial incentive of 22,500 TZS (~$10 USD) monthly for up to 6 months, conditional on HIV clinic visit attendance. A previously described constrained randomization approach [47] was used due to the small number of clinics in the study and to ensure that the study arms were balanced on key covariates (geographic region, facility level, driving distance to a major city, proximity to a major road, mean number of ART initiates per quarter).

All patients at participating study clinics meeting the following eligibility criteria were eligible to participate: (1) 18 years of age; (2) living with HIV infection (3) initiated ART less than or equal to 30 days prior to enrollment in the study; (4) had access to a mobile phone; and (5) did not intend to transfer to a different facility for HIV care within the next 12 months.

Participants could receive up to ~$60 over the 6-month intervention period if all monthly clinic visits were attended. The incentive size was selected based on two previous studies; the first study showed that there were no significant differences in ART retention and adherence between individuals receiving incentives (~$10 USD) and those receiving similarly valued food baskets, and that participants preferred monetary incentives over food [24]. The second study compared the effectiveness of two incentive amounts (~$5 and ~$10 USD) and found that the larger incentive amount had a greater effect on treatment retention and viral suppression than the smaller amount.^12^ The intervention was delivered using a mobile technology health (mHealth) system; patients presenting to the clinic were identified using biometric data (fingerprints) or their clinical ID number and the incentive was directly transferred via mobile money. Participant enrollment began in May 2021; 6-month follow-up was completed in November 2022 and comprise the data used for this analysis.

### Procedures

A survey module focused on IPV was administered in Kiswahili by trained research assistants on tablets using Qualtrics [49] to all female participants who reported being in a relationship. The survey module included all IPV-related questions from the World Health Organization Multi-Country Study on Women’s Health and Domestic Violence Against Women questionnaire [50]. Participants were offered a referral sheet with information about local services (medical, psychosocial, legal) for survivors of violence and had the option of leaving it with the research assistant to reduce any unintended risks of violence that could arise if others were to learn of their study participation.

### Outcomes

The primary outcome for this analysis is a binary indicator of past 6-month IPV measured 6 months after enrollment into the parent trial. IPV was defined as any experience of physical, sexual, and/or emotional violence, including controlling behaviors, by an intimate partner. We examined each type of IPV individually as secondary outcomes.

### Sample Size and Power

The parent trial investigating the financial incentive-viral suppression relationship had 80% power for a minimum detectable effect of 11 percentage points at 12 months, as an absolute increase in the proportion of virally suppressed participants in the incentive arm. With a fixed sample size at enrollment and 513 women reporting relationships at baseline with a prevalence of 62% past 6-month IPV, our analysis was powered at 80% (alpha = 0.05, two-tailed test) for a minimum detectable effect of the financial incentive on past 6-month IPV of at least 17 percentage points (i.e., decrease from 62 to 45% IPV in the incentive group).

### Statistical Analysis

Descriptive statistics were calculated to compare sociodemographic characteristics of female study participants by study arm at baseline using chi-square and t-tests and an alpha of 0.05. The prevalence of past 6-month IPV was assessed at baseline and at 6 months.

An intention-to-treat (ITT) analysis was conducted to examine the effect of financial incentives on past 6-month IPV among females who reported a partnership at 6 months. Unadjusted prevalence differences (PD) and 95% confidence intervals (CI) were estimated using generalized estimating equations (binomial family, identity link) with an exchangeable working correlation accounting for clustering by clinic site and robust standard errors. Adjusted models controlled for the duration of the relationship (partnership at baseline and 6 months vs. new partnership since baseline). We used inverse probability of censoring weighting (IPCW) to account for missing outcomes at 6 months.

Two subgroup analyses were conducted among: (1) female participants who were in a relationship at 6 months *only* (no relationship at baseline); and (2) female participants who reported being in a relationship both at baseline *and* 6 months. Adjusted models controlled for baseline reports of past 6-month physical IPV in the second subgroup (relationship at baseline and 6 months), as there were meaningful differences in physical IPV by study arm at baseline. Estimates were compared across models to explore whether there were differential effects of cash on IPV across subgroups, as introducing a new financial dynamic into a partnership may affect women in newer relationships differently than those who have been in relationships for > 6 months.

## Results

Between May 2021 and March 2022, 1,186 female participants were enrolled in the trial (*n* = 1,996 adult participants overall), of which 513 (43.4%) were partnered at baseline (married or boyfriend) (Supplementary Table S1). Among partnered women at baseline, 346 (67.4%) reported a lifetime experience of IPV and 320 (62.4%) reported experiencing IPV in the 6 months prior to the baseline survey (Supplementary Table S2). Emotional violence was the most common form of IPV, experienced by 313 (61.0%) participants in the past 6 months. Past 6-month physical and sexual violence were reported by 47 (9.2%) and 34 (6.6%) participants, respectively.

The analytic sample included 494 (41.7%) women who reported being in a partnership at 6 months, 307 (62.1%) of which were also partnered at baseline and 185 (37.5%) who reported partnerships at 6 months only. At baseline, participants were 33 years on average and 282 (57.1%) had completed primary school. Reports of all IPV measures were higher in the incentive arm than in the SOC when restricting to the 307 partnered women at baseline who received questions about violence, particularly the prevalence of past 6-month physical IPV (5.3% SOC vs. 11.5% incentive); however, these differences were not statistically significant (Table 1).

**Table 1:** Baseline demographics of female study participants in a relationship at 6 months (analytic sample) in regions in the Lake Zone, Tanzania, 2021

	SOC (*n* = 236)	Incentive (*n* = 258)	*p*-value	Overall (*n* = 494)
Age (years)				
Mean ± SD	33 ± 9.6	33 ± 11	0.546	33 ± 10
Median (IQR)	31 (26, 38)	30 (25, 39)		30 (26, 39)
**Partnered**	150 (63.6%)	157 (60.9%)	0.516	307 (62.1%)
Lifetime IPV*	104 (69.3%)	113 (72.0%)	0.787	217 (70.7%)
Past 6-month IPV*	93 (62.0%)	103 (65.6%)	0.590	196 (63.8%)
Past 6-month physical IPV*	8 (5.3%)	18 (11.5%)	0.082	26 (8.5%)
Past 6-month sexual IPV*	6 (4.0%)	10 (6.4%)	0.492	16 (5.2%)
Past 6-month emotional IPV*	92 (61.3%)	99 (63.1%)	0.613	191 (62.2%)
**Worked in the past 7 days**	141 (59.7%)	157 (60.9%)	0.974	298 (60.3%)
**Completed primary school**	130 (55.1%)	152 (58.9%)	0.586	282 (57.1%)
**Farming as main employment**	150 (63.6%)	163 (63.2%)	0.905	313 (63.4%)
**Household hunger**				
Little to no hunger	218 (92.4%)	238 (92.2%)	0.390	456 (92.3%)
Moderate hunger	15 (6.4%)	18 (7.0%)		33 (6.7%)
Severe hunger	0 (0.0%)	2 (0.8%)		2 (0.4%)
**Kiswahili as primary language**	80 (33.9%)	94 (36.4%)	0.674	174 (35.2%)

Missing data for variables: partnership status (*n* = 2), completed primary school (*n* = 8), worked in the past 7 days (*n* = 6), farming as main employment (*n* = 2), household hunger (*n* = 3), Kiswahili as primary language (*n* = 6)

*Reported only among women partnered at baseline (overall *n* = 307, SOC *n* = 150, incentive *n* = 157)

IPV: intimate partner violence; SOC: standard of care; SD: standard deviation; IQR: interquartile range

Physical IPV includes behaviors such as slapping, pushing, shoving, hitting, kicking, dragging, choking, and threatening with a weapon. Sexual IPV includes being physically forced to have sexual intercourse, having sexual intercourse unwillingly due to fear, and being forced to do something sexual that was degrading or humiliating. Emotional violence includes being yelled at, insulted, belittled, humiliated, intimidated, or threatened – or experiencing controlling behaviors by a partner such as being restricted or needing permission to see friends, family, or healthcare providers, being ignored, or suspicion about unfaithfulness

### Past 6-month IPV at 6 Months

At the end of the 6-month intervention period, 241 of 494 (48.8%) partnered women reported that they had experienced IPV in the past 6 months. Emotional IPV remained the most common form of violence at 6 months, reported by 48.8% of partnered women overall, followed by physical (7.5%) and sexual IPV (5.1%). Reports of IPV were modestly higher in the SOC arm compared to the incentive arm (49.6% SOC vs. 48.1% incentive), however there were no significant differences in violence across study arms, both in the unadjusted models and when controlling for relationship duration (PD: -0.02, 95% CI: -0.18, 0.15; adjusted PD [_a_PD]: -0.02, 95% CI: -0.19, 0.14) (Table 2).

**Table 2:** Effect of financial incentives on past 6-month intimate partner violence (emotional, physical, and sexual violence) among all women in relationships at 6 months

All women in relationships at 6 months						
	SOC (*n* = 236)	Incentive (*n* = 258)	Unadjusted PD (95% CI)	Adjusted PD (95% CI)	Unadjusted IPCW PD (95% CI)	Adjusted IPCW PD (95% CI)
**IPV (overall)**	117 (49.6%)	124 (48.1%)	-0.02 (-0.18, 0.15)	-0.02 (-0.19, 0.14)	-0.01 (-0.18, 0.15)	-0.02 (-0.18, 0.15)
**Emotional IPV**	114 (48.3%)	122 (47.3%)	-0.02 (-0.18, 0.15)	-0.02 (-0.19, 0.14)	-0.01 (-0.18, 0.15)	-0.01 (-0.18, 0.15)
**Physical IPV**	13 (5.5%)	24 (9.3%)	0.03 (-0.02, 0.09)	0.03 (-0.02, 0.09)	0.03 (-0.03, 0.09)	0.03 (-0.01, 0.09)
**Sexual IPV**	10 (4.2%)	15 (5.8%)	0.02 (-0.02, 0.06)	0.01 (-0.03, 0.04)	0.02 (-0.02, 0.06)	0.01 (-0.03, 0.04)

Prevalence differences and 95% CIs generated using generalized estimating equations (binomial family and identity link) with an exchangeable working correlation accounting for clustering by clinic and robust standard errors

Adjusted models include a binary indicator of relationship duration (partnership at baseline *and* 6 months vs. new partnership since baseline)

Missing data for variables: IPV overall (*n* = 15 SOC, *n* = 17 incentive); emotional IPV (*n* = 16 SOC, *n* = 16 incentive); physical IPV (*n* = 8 SOC, *n* = 11 incentive); sexual IPV (*n* = 8 SOC, *n* = 10 incentive)

IPV: intimate partner violence; SOC: standard of care; CI: confidence interval; IPCW: inverse probability of censoring weighting

There were also no differences between study arms when examining each form of violence individually. The SOC arm had a qualitatively higher prevalence of emotional IPV (PD: -0.02, 95% CI: -0.18, 0.15; _a_PD: -0.02, 95% CI: -0.19, 0.14), while the incentive arm had a higher prevalence of physical (PD: 0.03, 95% CI: -0.02, 0.09; _a_PD: 0.03, 95% CI: -0.02, 0.09) and sexual violence (PD: 0.02, 95% CI -0.02, 0.06; _a_PD: 0.01, 95% CI: -0.03, 0.04). There were no significant differences between arms in the analyses controlling for baseline reports of past 6-month physical violence among women partnered at both baseline and 6 months (Supplementary Table S3).

Controlling behaviors were the most pervasive expression of emotional violence. Of the 236 women who experienced emotional IPV in the past 6 months, 190 (80.1%) reported controlling behaviors only (restricted ability to see friends, family, or healthcare providers, being ignored, suspicion), while 46 (19.5%) reported additional forms of emotional violence (being yelled at, insulted, belittled, humiliated, intimidated, or threatened).

### Subgroup Analyses

The subgroup analyses demonstrated no significant effects of financial incentives on IPV (Table 3). When restricting the analysis to the 185 women who reported new partnerships at 6 months, the prevalence of IPV overall and emotional violence was nonsignificantly higher in the SOC arm than the incentive arm (IPV overall: 47.6% SOC vs. 40.6% incentive; emotional IPV 46.4% SOC vs. 39.6% incentive), while physical violence was higher in the incentive arm (4.8% SOC vs. 7.9% incentive) and sexual violence was relatively consistent between arms (2.4% SOC vs. 2.0% incentive). Among the 307 women partnered at both baseline and 6 months, reports of violence in the past 6 months were qualitatively higher in the financial incentive arm compared to SOC (IPV overall: 50.7% SOC vs. 52.9% incentive; emotional IPV: 49.3% SOC vs. 52.2% incentive, physical IPV: 6.0% SOC vs. 10.2% incentive; and sexual IPV: 5.3% SOC vs. 8.3% incentive).

**Table 3:** Effect of financial incentives on past 6-month intimate partner violence (emotional, physical, and sexual violence) among women in relationships at 6 months (only) and women in relationships at baseline and 6 months

Among women in new relationships at 6 months (not partnered at baseline)						
	SOC (*n* = 84)	Incentive (*n* = 101)	Unadjusted PD (95% CI)	Adjusted PD (95% CI)	Unadjusted IPCW* PD (95% CI)	Adjusted IPCW PD (95% CI)
**IPV (overall)**	40 (47.6%)	41 (40.6%)	-0.04 (-0.25, 0.16)	-	-0.06 (-0.26, 0.15)	-
**Emotional IPV**	39 (46.4%)	40 (39.6%)	-0.05 (-0.25, 0.15)	-	-0.06 (-0.26, 0.14)	-
**Physical IPV**	4 (4.8%)	8 (7.9%)	0.03 (-0.04, 0.10)	-	0.03 (-0.04, 0.10)	-
**Sexual IPV**	2 (2.4%)	2 (2.0%)	0.01 (-0.05, 0.04)	-	0.00 (-0.05, 0.04)	-

Among women in relationships at baseline *and * 6 months only						
	SOC (*n* = 150)	Incentive (*n* = 157)	Unadjusted PD (95% CI)	Adjusted PD (95% CI)	Unadjusted IPCW* PD (95% CI)	Adjusted IPCW PD (95% CI)
**IPV (overall)**	76 (50.7%)	83 (52.9%)	0.05 (-0.13, 0.24)	-	0.05 (-0.13, 0.24)	-
**Emotional IPV**	74 (49.3%)	82 (52.2%)	0.06 (-0.13, 0.24)	-	0.06 (-0.24, 0.13)	-
**Physical IPV**	9 (6.0%)	16 (10.2%)	0.04 (-0.04, 0.13)	-	0.04 (-0.04, 0.13)	-
**Sexual IPV**	8 (5.3%)	13 (8.3%)	0.03 (-0.03, 0.09)	-	0.03 (-0.04, 0.05)	-

Prevalence differences and 95% CIs generated using generalized estimating equations (binomial family and identity link) with an exchangeable working correlation accounting for clustering by clinic and robust standard errors

Subgroup analyses did not adjust for relationship duration

Weights for IPCW model for women in relationships at 6 months only did not include living with a partner, lifetime IPV, and past 6-month IPV at baseline

Missing data for variables: IPV overall (*n* = 15 SOC, *n* = 17 incentive); emotional IPV (*n* = 16 SOC, *n* = 16 incentive); physical IPV (*n* = 8 SOC, *n* = 11 incentive); sexual IPV (*n* = 8 SOC, *n* = 10 incentive)

IPV: intimate partner violence; SOC: standard of care; CI: confidence interval; IPCW: inverse probability of censoring weighting

## Discussion

This study sought to examine the relationship between financial incentives and IPV among women initiating ART in Tanzania to understand how this form of economic support impacts women living with HIV, who experience high risk of IPV compared to the general population. Our findings indicate that nearly 70% of women in relationships had experienced physical, sexual, or emotional violence in their lifetime at baseline and more than 60% had a violent experience with a partner in the past 6 months. At the end of the 6-month incentive period, there were no significant differences in IPV among women in relationships in the financial incentive arm compared to the SOC, indicating that short-term, monthly incentives of $10 USD neither mitigated nor exacerbated risks of IPV in this study population when provided in the context of HIV care.

There was also no association found among women who were in longer partnerships compared to those who had initiated a relationship within the past 6 months. The findings from this exploratory analysis suggest that the duration of a relationship does not significantly affect whether a woman experiences IPV after receiving cash; however, our data show some qualitative differences between women in relationships of different durations, including a lower prevalence of past 6-month IPV in the financial incentive arm vs. SOC among women in newer relationships compared to their counterparts in relationships for 6 months or more. While these changes were not significant at the *p* < 0.05 level, the differing directions and patterns of the effect estimates may possibly indicate that women in longer partnerships are affected differently by incentives than those who initiated relationships more recently, and further research is warranted to examine how relationship duration may alter the impact of financial incentives on experiences of violence.

This study has important implications for the design and implementation of future interventions providing economic support to women living with HIV in low and middle income countries [51]. Our results indicate that small, short-term financial incentives do not pose significant harms for women initiating ART. To our knowledge, there are no studies that have explored the relationship between financial incentives and IPV during the sensitive period after HIV diagnosis and treatment initiation which can increase susceptibility to violence [7, 52, 53]. This study contributes to this knowledge gap by providing insight into how women are uniquely affected when economic support is introduced into the household under these vulnerable circumstances. This is especially important given the mounting evidence that women in violent relationships have lower access to HIV services and treatment and need innovative approaches to encourage and strengthen linkages to care [10], and as economic support programs are growing in popularity and in number, especially to improve outcomes among people living with HIV. Indeed, a study examining cash transfer interventions found that by 2014, 40 countries in sub-Saharan Africa had at least one unconditional cash transfer program, up from 21 in 2010 [29]. The number of programs likely exceed this estimate now, and is higher when including conditional financial incentive interventions.

Our results also contribute to the growing body of literature suggesting that the effect of economic support on IPV is heterogeneous depending on the study population, and is influenced by many factors including the design, amount, delivery mechanism, and geographical area and sociocultural context in which interventions are implemented [29, 30, 35, 36, 54]. The financial incentive in this study was a conditional cash transfer of ~$10 USD dispersed monthly for up to six months for individuals initiating HIV treatment. Notably, this incentive mechanism, which was designed to subsidize costs that are commonly reported as barriers to HIV care (e.g., transportation, food) and motivate ART adherence via a small behavioral nudge, is different than the types of economic support that have been shown to be beneficial and protective against IPV in other studies [29, 31, 32, 34, 55]. Many of the interventions that have shown positive effects have provided larger sums to households for longer periods as part of social safety net or anti-poverty programs and may have shifted the household financial dynamics in ways that are unattainable with an incentive of this size and duration. A monthly incentive of ~$10 USD may be insufficient to relieve household stress related to poverty or improve a woman’s bargaining power or financial autonomy – mechanisms hypothesized to reduce IPV.

While we did not find evidence of any negative effects of financial incentives, our data show that the incentive arm had a nonsignificant but consistently higher prevalence of physical and sexual IPV compared to the SOC at 6 months. This may be an artifact of baseline imbalance given that participants in the incentive arm reported double the prevalence of physical violence than the SOC prior to receiving the intervention. Nonetheless, it remains vital to exercise continued vigilance and to engage directly with beneficiaries in future programs to ensure that incentives do not pose any unknown risks to their safety at any point during the intervention period, as we were underpowered to detect smaller, but important differences across study arms. This is further supported by findings from previous qualitative research in a similar study population which showed that while small financial incentives do not alter IPV risks for most female beneficiaries, continuous consultations are recommended to assess whether additional safeguards are needed for the subset of women who do experience some forms of violence and/or negative backlash [56]. 

The study has several strengths. The parent trial used a rigorous randomized design and included nearly 2,000 people living with HIV across four regions in Tanzania. Our analysis leveraged the random assignments to provide causal estimates of IPV while employing best practices for gender-based violence research. However, there are some important limitations that should be noted. The survey was designed to ask IPV questions only among partnered women to limit undue burden for non-partnered participants. For this reason, we do not have estimates on IPV prevalence for the entire study population – only women who identified a partnership at the time of their survey. Given that relationship status is subjective and dynamic, it is possible that our estimates exclude women who had a romantic partner but did not consider themselves to be formally in a relationship, as well as women who were in a relationship at baseline and left their relationship over the course of the intervention period but before completing the 6-month survey. Importantly, the latter group could include women who ended their relationships due to financial incentive related violence (in cases where incentives exacerbated risks) and/or women who were motivated by the incentives or had greater ability to leave potentially violent partners (in cases where incentives mitigated risks). To address this, future studies should examine IPV among all female intervention beneficiaries regardless of reported relationship status. Lastly, our sample size was fixed based on enrollment and retention in the parent trial, and the width of the confidence intervals on our estimates suggest that the sample size may have limited our power to detect small or moderate effects.

In conclusion, we found that providing small, short-term incentives to women to motivate engagement in HIV care does not heighten risks of IPV nor provide any substantial protective benefits for IPV prevention. Further research is needed to assess their effects on larger samples as financial incentive and cash transfers programs are brought to scale for people living with HIV.

## Electronic Supplementary Material

Below is the link to the electronic supplementary material.

Supplementary Material 1

## Data Availability

De-identified participant data used in these analyses will be made available upon request.
