# Supplementary material for: Impact of Financial Incentives on Intimate Partner Violence for Women Living with HIV Initiating Antiretroviral Therapy
Source: AIDS Behav. 2025 Feb 17;29(5):1507–15. doi: 10.1007/s10461-025-04621-1 (PMC12031819; doi:10.1007/s10461-025-04621-1)
Supplement: Supplementary file 1 — Supplementary Material 1 [file 10461_2025_4621_MOESM1_ESM.docx]

**Supplementary Table S1. Baseline demographics of all female study participants in the trial, by study arm**

|  | **SOC**  **(n=533)** | **Incentive**  **(n=653)** | **p-value** | **Overall**  **(n=1186)** |
| --- | --- | --- | --- | --- |
| **Age (years)** |  |  |  |  |
| Mean ± SD | 34 ± 11 | 34 ± 11 | 0.691 | 34 ± 11 |
| Median (IQR) | 32 (26, 40) | 32 (25, 40) |  | 32 (26, 40) |
| **Partnered** | 255 (47.8%) | 258 (39.5%) | 0.003 | 513 (43.3%) |
| **Lives with partner** | 203 (38.1%) | 208 (31.9%) | 0.86 | 411 (34.7%) |
| **Lifetime IPV*** | 172 (32.3%) | 174 (26.6%) | 1.00 | 346 (29.2%) |
| **Past 6-month IPV*** | 159 (29.7%) | 161 (24.7%) | 0.063 | 320 (27.0%) |
| Past 6-month physical IPV | 17 (3.2%) | 30 (4.6%) | 0.072 | 47 (4.0%) |
| Past 6-month sexual IPV | 12 (2.3%) | 22 (3.4%) | 0.115 | 60 (5.1%) |
| Past 6-month emotional IPV | 154 (28.9%) | 159 (24.3%) | 0.777 | 313 (26.4%) |
| **Completed primary school** | 297 (55.7%) | 377 (57.7%) | 0.818 | 674 (56.8%) |
| **Worked in the past 7 days** | 289 (54.2%) | 411 (62.9%) | 0.006 | 700 (59.0%) |
| **Farming as main employment** | 341 (64.0%) | 386 (59.1%) | 0.061 | 727 (61.3%) |
| **Household hunger** |  |  |  |  |
| Little to no hunger | 491 (92.1%) | 597 (91.4%) | 0.231 | 1088 (91.7%) |
| Moderate hunger | 33 (6.2%) | 47 (7.2%) |  | 80 (6.7%) |
| Severe hunger | 2 (0.4%) | 8 (1.2%) |  | 10 (0.8%) |
| **Kiswahili as primary language** | 188 (35.3%) | 255 (39.1%) | 0.267 | 443 (37.4%) |
| Missing data for variables: partnership status (n=7), completed primary school (n=20), worked in the past 7 days (n=16), farming as main employment (n=7), Household hunger (n=8), Kiswahili as primary language (n=16)  IPV: intimate partner violence; SOC: standard of care; SD: standard deviation; IQR: interquartile range  *Reported only among women partnered at baseline (overall n=307, SOC n=150, incentive n=157) | | | | |

**Supplementary Table S2. Reports of intimate partner violence among partnered participants at baseline**

|  | **SOC**  **(N=255)** | **Incentive**  **(N=258)** | **Overall**  **(N=513)** |
| --- | --- | --- | --- |
| **Lifetime intimate partner violence (IPV)** | 172 (67.5%) | 174 (67.4%) | 346 (67.4%) |
| **Past 6-month IPV** | 157 (61.6%) | 163 (63.2%) | 320 (62.4%) |
| Past 6-month physical IPV | 17 (6.7%) | 30 (11.6%) | 47 (9.2%) |
| Past 6-month sexual IPV | 12 (4.7%) | 22 (8.5%) | 34 (6.6%) |
| Past 6-month emotional IPV | 154 (60.4%) | 159 (61.6%) | 313 (61.0%) |
| Missing data for variables: lifetime IPV (n=27)  IPV: intimate partner violence; SOC: standard of care |  |  |  |

**Supplementary Table S3. Adjusted effects of financial incentives on past 6-month intimate partner violence controlling for baseline reports of past 6-month physical intimate partner violence for women in relationships at baseline and 6 months**

| **Among women in relationships at baseline *and* 6 months only** | | | | | | |
| --- | --- | --- | --- | --- | --- | --- |
|  | **SOC**  **(n=150)** | **Incentive**  **(n=157)** | **Unadjusted**  **PD (95% CI)** | **Adjusted**  **PD (95% CI)** | **Unadjusted IPCW**  **PD (95% CI)** | **Adjusted IPCW**  **PD (95% CI)** |
| **IPV overall** | 76 (50.7%) | 83 (52.9%) | 0.05  (-0.13, 0.24) | 0.05  (-0.14, 0.23) | 0.05  (-0.13, 0.24) | 0.05  (-0.14, 0.23) |
| **Emotional IPV** | 74 (49.3%) | 82 (52.2%) | 0.06  (-0.13, 0.24) | 0.05  (-0.14, 0.24) | 0.06  (-0.13, 0.24) | 0.05  (-0.18, 0.24) |
| **Physical IPV** | 9 (6.0%) | 16 (10.2%) | 0.04  (-0.04, 0.13) | 0.04  (-0.04, 0.12) | 0.04  (-0.04, 0.13) | 0.04  (-0.04, 0.11) |
| **Sexual IPV** | 8 (5.3%) | 13 (8.3%) | 0.03  (-0.03, 0.09) | 0.03  (-0.03, 0.09) | 0.03  (-0.03, 0.09) | 0.03  (-0.03 0.09) |
| Prevalence differences and 95% CIs generated using generalized estimating equations (binomial family and identity link) with an exchangeable working correlation accounting for clustering by clinic and robust standard errors  Adjusted models include a binary indicator of past 6-month physical IPV at baseline  Missing data for variables: IPV overall (n=11 SOC, n=9 incentive); emotional IPV (n=12 SOC, n=9 incentive); physical IPV (n=6 SOC, n=8 incentive); sexual IPV (n=6 SOC, n=7 incentive), baseline physical IPV (n=6 SOC, n=7 incentive)  IPV: intimate partner violence; SOC: standard of care; CI: confidence interval; IPCW: inverse probability of censoring weighting | | | | | | |
